# Differential effects of intermittent energy restriction vs. continuous energy restriction combined high-intensity interval training on overweight/obese adults: A randomized controlled trial

**DOI:** 10.3389/fnut.2022.979618

**Published:** 2022-11-08

**Authors:** Rui Xu, You-Xiang Cao, Yu-Ting Chen, Yu-Qi Jia

**Affiliations:** ^1^School of Sports and Health, Nanjing Sport Institute, Nanjing, China; ^2^Laboratory of Kinesiology, Nanjing Sport Institute, Nanjing, China; ^3^School of Kinesiology, Shanghai University of Sport, Shanghai, China

**Keywords:** intermittent energy restriction, continuous energy restriction, high-intensity interval training (HIIT), weight loss, overweight/obese adults

## Abstract

**Background:**

Intermittent energy restriction (IER) and continuous energy restriction (CER) are increasingly popular dietary approaches used for weight loss and overall health. These energy restriction protocols combined with exercise on weight loss and other health outcomes could achieve additional effects in a short-term intervention.

**Objectives:**

To evaluate the effects of a 4-week IER or CER program on weight, blood lipids, and CRF in overweight/obese adults when combined with high-intensity interval training (HIIT).

**Methods:**

Forty-eight overweight/obese adults [age: 21.3 ± 2.24 years, body mass index (BMI): 25.86 ± 2.64 kg⋅m^–2^] were randomly assigned to iER, cER, and normal diet (ND) groups (*n* = 16 per group), each consisting of a 4-week intervention. All of the groups completed HIIT intervention (3 min at 80% of V̇O_2m*ax*_ followed by 3 min at 50% of V̇O_2m*ax*_), 30 min/training sessions, five sessions per week. iER subjects consumed 30% of energy needs on 2 non-consecutive days/week, and 100% of energy needs on another 5 days; cER subjects consumed 70% of energy needs; and ND subjects consumed 100% of energy needs. Body composition, waist circumference (WC) and hip circumference (HC), triglyceride (TG), total cholesterol (TC), low-density lipoprotein-cholesterol (LDL-c), high-density lipoprotein-cholesterol (HDL-c), and cardiorespiratory fitness (CRF) were measured before and after the intervention.

**Results:**

Of the total 57 participants who underwent randomization, 48 (84.2%) completed the 4-week intervention. After intervention body composition and body circumference decreased in three groups, but no significant differences between groups. The iER tends to be superior to cER in the reduction of body composition and body circumference. The mean body weight loss was 4.57 kg (95% confidence interval [CI], 4.1–5.0, *p* < 0.001) in iER and 2.46 kg (95% CI, 4.1–5.0, *p* < 0.001) in iER. The analyses of BMI, BF%, WC, and HC were consistent with the primary outcome results. In addition, TG, TC, HDL-c, and CRF improved after intervention but without significant changes (*p* > 0.05).

**Conclusion:**

Both IER and CER could be effective in weight loss and increased CRF when combined with HIIT. However, iER showed greater benefits for body weight, BF%, WC, and HC compared with cER.

## Introduction

Energy restriction feeding protects from diet-induced obesity as a result of reduced energy intake and increased fat oxidation (1, 2). Intermittent energy restriction (IER) and continuous energy restriction (CER) have received considerable recent interest as dietary restriction strategies for weight loss and improving glucose and lipid metabolism (3–5). Although IER and CER were both restricted diets, implementation details vary greatly. The CER diet is a daily calorie restriction, which reduces energy intake by a small amount (e.g., 25–30%) each day (6, 7). In contrast, the IER diet involves extended time periods (e.g., 16–48 h) with little or no energy intake, with intervening periods of *ad libitum* intake; or alternate day fasting, with little energy intake (e.g., 25–30%) 2 days per week and *ad libitum* intake for the other 5 days (8–10). Studies in rodents shown that fasting glucose and insulin were improved after both IER and CER intervention (11, 12). However, the relative contributions of IER and CER protocols for weight loss are controversial. Previous studies reported that IER and CER have the equivalent effect on body weight (BW) loss (13), but showed different effects on body composition (14–16) and glycolipid metabolism (4, 16).

Although dietary restriction results in weight loss, it was accompanied by loss of muscle and reduced health fitness (17), dietary restriction combined with exercise is an effective strategy in weight loss management for overweight and obese adults (18, 19). The addition of specific exercise training to energy restriction in obesity may, in addition to improve physical fitness, also helps in body composition (20). Exercise combined with dietary restriction was the most effective way to lose weight and maintain a modest weight long term for individuals with obesity (21), it could reduce more calories and push the body further into negative energy balance (22), while IER and CER combined with exercise could be greater than the mere sum of the parts. Several important questions remain to be answered, including whether the weight loss effect varies when the dietary restriction combined with exercise in overweight/obese adults, and which aspect was the most effective for improvement. Under conditions of altered glycolipid metabolism with different types of energy restriction, the energy supply available for exercise varies between IER and CER.

High-intensity interval training (HIIT) interventions have emerged in recent years as a time-efficient means to reduce body weight and improve cardiorespiratory fitness (CRF) in the short-term intervention (23–25), especially for the young obese population without cardiovascular disease (26, 27). It can achieve similar or even superior changes in physiological and physical performance and health-related outcomes than moderate-intensity continuous training in overweight/obese adults with less training time (28, 29).

Given the above, this study was conducted as 4-week randomized control dietary restriction and HIIT intervention in overweight/obese adults. We aimed to explore whether the combination of a caloric restriction dietary program and HIIT could improve the effectiveness. We also sought to compare the effects of IER vs. CER on body composition, body circumference, blood lipids, and CRF.

We hypothesized that the caloric restriction protocol combined with HIIT was more effective for weight loss than HIIT intervention only and that the intermittent caloric restriction protocol would lead to greater fat loss and improvements in lipid metabolism-related biomarkers compared to continuous energy restriction.

## Subjects and methods

### Study design and participants

#### Study design

**FIGURE 1:**
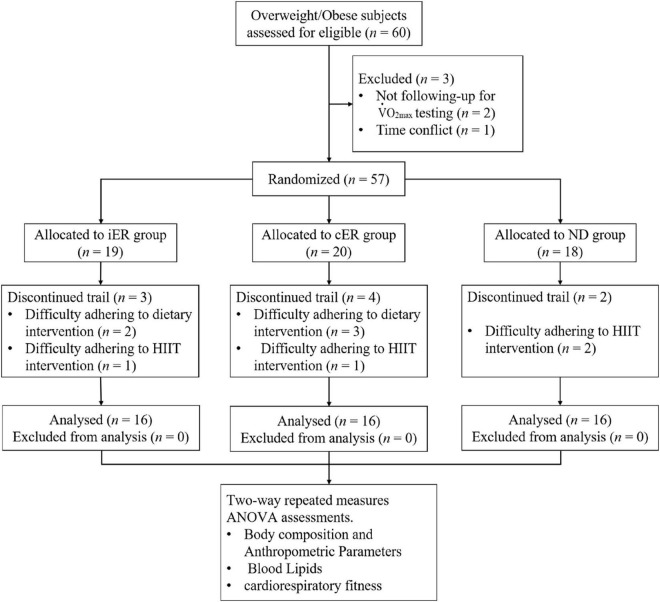
Consort flow diagram. This figure illustrates the number of participants from enrollment to completion of the trial. A total of 60 overweight/obese subjects were deemed eligible and consented to partake in the study. Two participants dropped out due to not followed $\dot{\text{V}}\,{\text{O}}_{2}$_*max*_ testing, and 1 participant had a time conflict with exercise training. After completing the baseline period, participants were randomized to an iER group (*n* = 19), a cER group (*n* = 20), or a ND group (*n* = 18). During the 4-week intervention, nine participants removed themselves from the study due to could not adhere to dietary intervention or HIIT intervention, but none discontinued due to adverse events. Thus, totally 48 participants (*n* = 16 in each group) completed the study.

We conducted a randomized controlled trial, in which participants were randomized to iER group (intermittent energy restriction dietary combined with HIIT), cER group (continuous energy restriction dietary with HIIT), and ND group (normal dietary with HIIT) (Figure 1). The study design and experimental protocol were approved by the Nanjing Sports Institute Laboratory Ethics Committee (No. RT202102).

#### Study participants

60 participants who were overweight (body mass index [BMI] ≥ 24 kg/m^2^) or obese (BMI ≥ 28 kg/m^2^) (30) aged 18–30 years were recruited from the university. Participants who had secondary obesity or were at high risk of cardiovascular and diabetes were excluded based on a modified American Heart Association/American College of Sports Medicine (ACSM) health/fitness facility pre-participation questionnaire (31, 32). Exclusion criteria were as follows: type 1 or 2 diabetes; any significant systemic disease or disorder including malignancy, inflammatory, or endocrine conditions; pregnancy or breastfeeding; BMI < 24 kg/m^2^; systolic blood pressure (SBP) > 160 mmHg or diastolic blood pressure (DBP) > 100 mmHg. Additionally, participants were screened *via* questionnaire to ensure that they had normal dietary habits and were not consuming other nutritional supplements or medications which would affect metabolism and body weight (33). Once participants had chosen which diet protocol they wished to follow, random assignment to an intervention arm (control or 1 of 3 monitoring strategies) was performed using sequentially numbered, opaque, sealed envelopes prepared by the statistician, stratified by sex and random-length blocks. We excluded three subjects before the intervention and nine subjects who quit during the intervention (three from the iER group, four from the cER group, and two from the ND group). Finally, totally 48 subjects were included in the study. We obtained written informed consent from each participant before screening and data collection. All the methods were conducted in accordance with the approved guidelines and regulations.

### Diet intervention

The estimated energy requirements were calculated using the factorial approach. We used the Henry predictive equation: daily energy needs = basal metabolic rate (BMR) × physical activity level (PAL) (34), which takes into account the effects of variations in physical activity participation and BMR on daily energy needs. The ACSM physical activity questionnaire (PAR-Q +) (31) was used to investigate the physical activity of the subjects.

The BMR was estimated with the following equations: BMR = 51 × BW (for men aged 18–30years), 47 × BW (for women aged 18–30years) (35). PAL was graded according to the PAR-Q + questionnaire result from each subject and was defined as low active (1.55 for men and 1.56 for women), moderately active (1.78 for men and 1.64 for women), or vigorously active (2.10 for men and 1.82 for women) (36). The daily recommended energy intakes were 1,800–2,250 kcal for females and 2,250–2,700 kcal for males, as recommended by Dietary Guidelines for Chinese Residents Recommended Reference Intakes (36).

**FIGURE 2:**
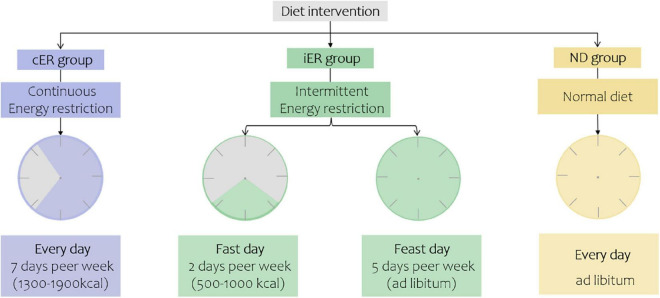
Diet intervention of three groups. Continuous energy restriction, 30% of their daily recommended energy intake (approximately 500–1000 kcal) on 2 non-consecutive days every week and consumed food *ad libitum* for the other 5 days to maintain their normal energy intake. Intermittent energy restriction, 70% of their estimated energy requirements every day. Normal diet, 100% of their energy needs to be divided into three meals.

**FIGURE 3:**
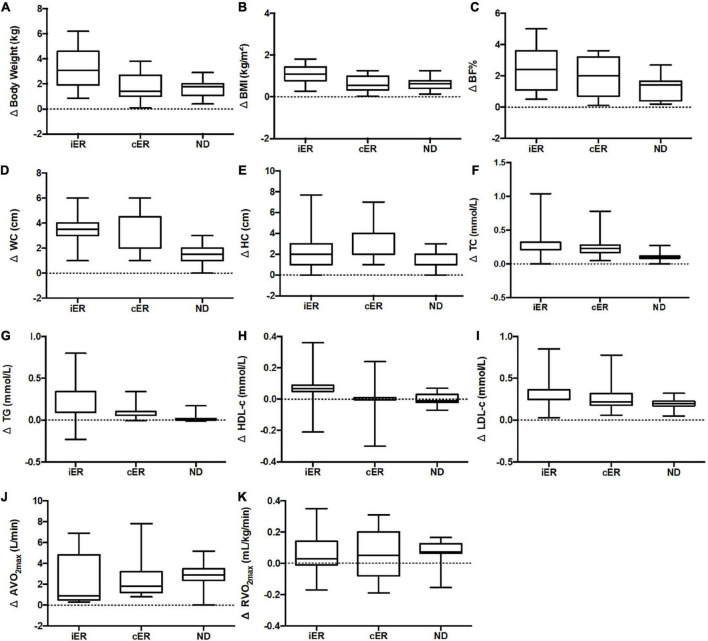
**(A)** Change in body weight after 4 weeks of intervention. **(B)** Change in BMI after 4 weeks of intervention. **(C)** Change in BF% after 4 weeks of intervention. **(D)** Change in WC after 4 weeks of intervention. **(E)** Change in HC after 4 weeks of intervention. **(F)** Change in TC after 4 weeks of intervention. **(G)** Change in TG after 4 weeks of intervention. **(H)** Change in HDL-c after 4 weeks of intervention. **(I)** Change in LDL-c after 4-week of intervention. **(J)** Change in AV̇O_2m*ax*_ after 4 weeks of intervention. **(K)** Change in RV̇O_2m*ax*_ after 4 weeks of intervention. *n* = 16 in each group. Delta (Δ), change from pre- post-intervention. Ier, intermittent energy restriction combined with HIIT group; cER, continuous energy restriction combined with HIIT group; ND, normal diet group combined with HIIT. BMI, body mass index; BF%, percentage body fat; WC, waist circumference; HC, hip circumference TC, total cholesterol; TG, triglyceride; LDL-c, low-density lipoprotein-cholesterol; HDL-c, high-density lipoprotein-cholesterol; AV̇O_2m*ax*_, absolute maximal oxygen uptake; RV̇O_2m*ax*_, the maximal oxygen uptake relative to body weight. Data is visualized as Tukey box plots (line at mean, top of the box at the 75th percentile, bottom of the box at the 25th percentile, whiskers at the highest and lowest values, outliers shown as triangles beyond the whiskers). Two-way repeated measures ANOVA used for results (sphericity assumed) could be between-group comparisons.

The iER and cER groups participated in the 4-week dietary intervention. iER subjects consumed 30% of their daily recommended energy intake (approximately 500–1,000 kcal) on 2 non-consecutive days every week and consumed food *ad libitum* for the other 5 days by their daily recommended energy intake. The cER subjects were advised to consume a daily hyperenergetic diet of 70% of their estimated energy requirements (approximately 1,300–1,600 kcal for females and 1,600–1,900 kcal for males). The dietary composition was provided in the daily dietary log by professional nutritionists. The total calorie intake was equivalent between the iER and cER groups each week. Subjects in the ND group consumed 100% of their daily recommended energy intake divided into three meals (Figures 2, 3).

Healthy eating advice (36) and individualized food portion lists were provided by an appropriately trained study investigator. The 24-h dietary recall method was used to collect data concerning food consumption by participants during the previous 24 h. Intake of total calories, protein (20–35%), carbohydrate (55–65%), and fat (10–15%) were computed using the Chinese Dietary Guidelines (2016) produced by the Chinese Nutrition Society (36).

During the 4-week intervention, the iER and cER groups were instructed to report their dietary logs daily, and each meal was recorded by taking photos on the subject’s cell phone. The WeChat-supported was used to assist with the supervision of the subjects during the experiment to help them to complete the study. One designated researcher who had got a kinesiology professional was trained to estimate the number of different types of food using the posted photos, which would be double-checked by another researcher. Standardized measurement guides were used to assess portion sizes. The records for all the meals of every participant were included in the analysis. Diet and exercise compliance was assessed by recording attendance. If a “fast day” or “an exercise session” missed, the subject was required to make up for the missed day on another day of the week.

#### Exercise intervention

HIIT exercise intervention was administered to 48 subjects who were randomly assigned to the iER, cER, or normal diet (ND) group (*n* = 16 per group). Each group was instructed to follow the HIIT intervention and their assigned diet protocol during the 4-week intervention period. Baseline measurements and post-intervention measurements were performed pre-and post-intervention. The parameters included body composition, body circumferences, blood lipids, and cardiorespiratory fitness tests. Two-way repeated measures ANOVA were carried out to evaluate the effect of the time (before and after intervention), group (intermittent energy restriction, continuous energy restriction, normal diet), and the time-group interaction. All of the groups used a HIIT protocol during the 4-week intervention. The maximum oxygen uptake of the subject was tested before the intervention to determine the subject’s maximum oxygen uptake level.

The subjects underwent exercise training with 80% V̇O_2m*ax*_ during high-intensity periods, separated by brief periods of low-intensity activity with 50% V̇O_2m*ax*_ of the individual, including five bouts of 3 min cycling, resulting in 30 min exercise/training session (29). Each exercise session began with 10 min of warm-up with 50% V̇O_2m*ax*_ and ended with 10 min of cool down. The HIIT protocol was designed to induce 310 kcal energy deficit and the duration of the exercise session was individually tailored. The frequency of exercise was 5 days per week for 4 weeks. Training for the IER group was arranged on the 5 *ad libitum* days.

During exercise, the heart rate of the subjects was monitored using Polar H10 heart rate sensor (Polar H10, Polar Electro Oy, Finland). The subjects were closely monitored for subjective fatigue by Borg’s Ratings of Perceived Exertion 6–20 to ensure that the trial was accurate and safe (37).

### Outcome measurements

All of the participants underwent outcome measured at baseline and after 24 h following the end of the study period. All of the measurements were taken by research assistants who were blinded to the intervention (monitoring strategy) and diet group allocation, to reduce the risk of bias and improve the methodological quality of randomized controlled trials (38). All of the measurements were performed by well-trained kinesiology professionals who completed the research program in accordance with standard operating procedures. All of the parameters were measured by the same tester pre-and post-intervention.

#### Body composition anthropometric assessments

We measured the body weight, percentage of body fat (BF%), and total lean mass using dual-energy X-ray absorptiometry scan (DXA) (GE Lunar Prodigy; GE Healthcare, Madison, WI, USA), accurate to 0.1 kg, 0.1%, respectively. The tests were conducted in the morning with an empty stomach and limited fluids state. The subjects were asked to dress in light clothing, remain quiet, without any physical activity until finish the test. The BMI was calculated as the ratio of weight (kg) to height squared (m^2^).

#### Body circumferences

Waist and hip circumferences were measured using an inelastic plastic fiber tape measure placed directly on the skin at the midpoint between the lower border of the rib cage and the iliac crest (for waist) and at the maximum extension of the buttocks (for hip). The measurements were recorded to the nearest 0.1 cm. The WHR was calculated as the ratio of the waist (cm) to the hip (cm).

#### Blood sampling

To assess blood metabolism parameters, we obtained blood samples from ulnar vein after an 8-h overnight fast in the hospital at the beginning and the end of the trial. Venous blood samples were collected from the arm into a 6-ml plasma separator tube (BD Vacutainer; Franklin Lakes, NJ, USA) using standard aseptic phlebotomy techniques. The plasma was extracted after centrifugation (30 min at 1,000 g) and stored at −80^°^C for measured. Plasma was analyzed for triglyceride (TG), total cholesterol (TC), low-density lipoprotein-cholesterol (LDL-c), and high-density lipoprotein-cholesterol (HDL-c) using an automated analyzer (Olympus AU5400, Japan). All of the instruments used in this study were calibrated before testing.

#### Cardiorespiratory fitness

Indirect measurement of maximal oxygen uptake was performed with Bruce incremental treadmill testing program on a treadmill (H/P/cosmos, Germany) with the metabolic analyzer K5 (COSMED, Rome, Italy). The test started with a 7 km/h warm-up, during which the speed was increased gradually by 1 km/h every 1 min until maximal speed. The criteria set for achieving the maximum oxygen uptake (V̇O_2m*ax*_) were any of the following: (1) age-adjusted maximal heart rate attained; (2) a plateau of oxygen uptake was attained, the oxygen uptake no longer increased accompanied with the increase of exercise intensity; (3) respiratory quotient (RQ) of over 1.15 (39). The heart rate was monitored throughout the testing program by heart rate sensor (Polar H10, Polar Electro Oy, Finland). Maximal heart rate was defined by adding five beats to the highest heart rate value obtained during the V̇O_2m*ax*_ test. The ratings of perceived exertion were recorded during the last 30 s of each workload (40). Research personnel provided verbal encouragement and support to assist participants in reaching a maximal effort. The termination criteria for the V̇O_2m*ax*_ test was any of the following: (1) angina pectoris or angina-like symptoms; (2) hyperelevation of blood pressure (BP), SBP>250 mmHg, and/or DBP>115 mmHg; (3) age-adjusted maximal heart rate attained; (4) a plateau of oxygen uptake was attained; (5) if the subject was unable to maintain the pace of the treadmill; (6) Respiratory Exchange Ratio (RER) of over 1.3 and/or a plateau in ventilation (41).

Participants were instructed not to eat or drink beverages other than water for at least 90 min before their treadmill test. Before the baseline test, participants practiced walking on the treadmill without holding onto the handrails. Before each test, participants were familiarized with the testing protocol.

### Statistical analysis

Statistical analysis was conducted using SPSS 25.0 (SPSS Inc., Chicago, IL). All of the data are presented as the mean ± standard deviation (SD) or number (%). Before performing the main statistical analyses, all variables were checked for normality using the Shapiro—Wilk test. To compare the three groups, two-way repeated measures ANOVA were used to evaluate the effect of the time (before and after intervention), group (intermittent energy restriction, continuous energy restriction, normal diet), and the time-group interaction. Mauchly’s Test of Sphericity was performed, and when the sphericity hypothesis was established, two-way repeated measures ANOVA results (Sphericity Assumed) could be used directly; when the sphericity hypothesis was not valid, the corrected results (Greenhouse-Geisser correction) were used. Bonferroni *post-hoc* tests were employed to locate specific differences. Partial eta-squared (η^2^*_*p*_*) was adopted for interactions: 0.001–0.059 represented a small effect, 0.06–0.139 a moderate effect and higher than 0.14 a large effect (42). Statistical significance was set at *p* < 0.05. We used chi-square test to compare the gender differences between groups.

## Results

### Dietary intake

The daily recommended energy intake of a normal diet in iER, cER, and ND were 2480.74 ± 603.30 kcal, 2472.66 ± 499.34 kcal, and 2601.63 ± 340.28 kcal. One-way ANOVA indicated no significant difference (*p* = 0.726) between the three groups. iER group consumed 30% of their daily recommended energy intake was 744.22 ± 180.99 kcal on the restricted dietary day which in 2 non-consecutive days every week and another 5 days keep their normal daily energy intake. cER group intake of 1730.86 ± 349.52 kcal every day during the 4-week intervention which consumed 70% of their daily recommended energy intake. ND group in accordance with daily recommended energy intake during the 4-week intervention. One-way ANOVA indicated a significant difference (*p* < 0.001) between three groups for total energy intake per week, ND group intake significant more energy per week compared with iER or cER groups (both *p* = 0.000), but parametric *post hoc* analysis indicated no significant difference between iER and cER (13892.16 ± 3378.47 kcal *vs.* 12116.02 ± 2446.78 kcal, *p* = 0.087) (Table 1).

**TABLE 1:** Dietary intake during the intervention.

	iER (*n* = 16)	cER (*n* = 16)	ND (*n* = 16)	*p*
Daily recommended energy intake (kcal)	2,481 ± 603	2,473 ± 499	2,602 ± 340	0.726
Restriction of dietary day intake (kcal)	744.2 ± 181	1730.9 ± 349	-	-
Total energy intake per week (kcal)	13,892 ± 3,379	12,116 ± 2,447	18,211 ± 2,382	<0.001

Values are presented as arithmetic means ± SD. iER, intermittent energy restriction group; cER, continuous energy restriction group; ND, normal diet group.

### Baseline characteristics of participants

Participants’ baseline characteristics are described in Table 2. There are no differences between groups.

**TABLE 2:** Baseline characteristics.

	iER (*n* = 16)	cER (*n* = 16)	ND (*n* = 16)	*F*	*p*
Sex (M/F)	7/9	6/10	8/8	-	0.776
BMI (kg⋅m^–2^)	26.90 ± 1.46	26.39 ± 1.63	26.12 ± 2.01	0.799	0.457
BF%	33.96 ± 4.81	33.47 ± 3.84	33.54 ± 3.48	0.063	0.939
Total lean mass (kg)	48.89 ± 13.85	48.61 ± 11.01	48.17 ± 6.15	0.017	0.983
WC (cm)	86.86 ± 7.95	86.79 ± 8.44	86.33 ± 5.93	0.075	0.927
HC (cm)	100.91 ± 6.70	99.83 ± 6.50	100.52 ± 3.01	0.139	0.871
WHR	0.86 ± 0.06	0.86 ± 0.47	0.86 ± 0.52	0.021	0.979
TC (mmol⋅L^–1^)	4.24 ± 0.29	4.23 ± 0.45	4.26 ± 0.12	0.042	0.959
TG (mmol⋅L^–1^)	1.09 ± 0.25	1.09 ± 0.27	1.05 ± 0.09	0.145	0.866
HDL-c (mmol⋅L^–1^)	1.39 ± 0.13	1.36 ± 0.08	1.38 ± 0.20	0.208	0.813
LDL-c (mmol⋅L^–1^)	2.39 ± 0.31	2.42 ± 0.19	2.33 ± 0.28	0.372	0.692
RV̇O_2m*ax*_ (mL/kg/min)	36.95 ± 11.91	40.23 ± 8.00	34.79 ± 6.11	1.418	0.254
AV̇O_2m*ax*_ (L/min)	2.73 ± 0.96	2.99 ± 0.50	2.59 ± 0.37	1.370	0.265
Physical activity level (METs/week)	1512.73 ± 185.11	1433.40 ± 271.90	1497.60 ± 288.62	0.417	0.662

Outcome variables are presented as means ± SD. M, male; F, female; iER, intermittent energy restriction combined with HIIT group; cER, continuous energy restriction combined with HIIT group; ND, normal diet combined with HIIT group. BMI, body mass index; BF%, percentage body fat; WC, waist circumference; HC, hip circumference; WHR, the waist-hip ratio; TC, total cholesterol; TG, triglyceride; LDL-c, low-density lipoprotein-cholesterol; HDL-c, high-density lipoprotein-cholesterol; AV̇O_2max_, absolute maximal oxygen uptake; RV̇O_2max_, relative maximal oxygen uptake.

### Body composition and anthropometric parameters

After two-way ANOVA with repeated measures, the interaction of group and time for body weight [*F*_(2, 28)_ = 91.833, *p* < 0.001, η^2^*_*p*_* = 0.868], BMI [*F*_(2, 28)_ = 104.405, *p* < 0.001, η^2^*_*p*_* = 0.882], BF% [*F*_(1_._374, 19_._231)_ = 21.836, *p* < 0.001, η^2^*_*p*_* = 0.2609], WC [*F*_(2, 28)_ = 8.412, *p* = 0.001, η^2^*_*p*_* = 0.375], HC [*F*_(2, 28)_ = 4.596, *p* = 0.019, η^2^*_*p*_* = 0.247] was significant (Table 3), and *post hoc* analyses indicated that there was a significant decrease in each group after intervention, but there was no difference by groups (Tables 4, 5).

**TABLE 3:** Two-way ANOVA with repeated measures in the main effect of body composition and anthropometric parameters.

	Time effect			Group effect			Interaction effect		
	*F*	*p*	η^2^*p*	*F*	*p*	η^2^*p*	*F*	*p*	η^2^*p*
BW (kg)	675.620	< 0.001	0.980	0.182	0.835	0.013	91.833	< 0.001	0.868
BMI (kg⋅m^–2^)	663.895	< 0.001	0.979	0.046	0.995	0.003	104.405	< 0.001	0.882
BF%	269.979	< 0.001	0.951	0.654	0.528	0.045	21.836	< 0.001	0.609
Total lean mass (kg)	0.005	0.943	0	0.006	0.994	0	2.514	0.099	0.152
WC (cm)	383.424	< 0.001	0.965	0.141	0.869	0.010	60.294	< 0.001	0.812
HC (cm)	157.243	< 0.001	0.918	0.399	0.675	0.028	40.066	< 0.001	0.741
WHR	1.535	0.236	0.099	0.003	0.997	0	1.429	0.257	0.093

BMI, body mass index; BF%, percentage body fat; WC, waist circumference; HC, hip circumference; WHR, the waist-hip ratio.

**TABLE 4:** Body composition and anthropometric parameters before and after intervention.

	iER			cER			ND		
	MD (95%CI)	*F*	*p*	MD (95%CI)	*F*	*p*	MD (95%CI)	*F*	*p*
BW (kg)	4.568 (4.130, 5.006)	499.689	< 0.001	2.463 (2.052, 2.874)	165.485	< 0.001	1.399 (1.152, 1.645)	147.937	< 0.001
BMI (kg⋅m^–2^)	1.650 (1.480, 1.820)	432.470	< 0.001	0.861 (0.736, 0.985)	219.870	< 0.001	0.487 (0.402, 0.573)	148.569	< 0.001
BF%	5.779 (5.103, 6.454)	336.436	< 0.001	3.147 (1.805, 4.490)	25.271	< 0.001	1.753 (1.368, 2.139)	95.244	< 0.001
WC (cm)	5.253 (4.565, 5.942)	267.599	< 0.001	2.273 (1.810, 2.763)	110.864	< 0.001	1.547 (1.068, 2.025)	48.039	< 0.001
HC (cm)	4.169 (4.291, 6.047)	159.415	< 0.001	2.487 (1.775, 3.198)	56.220	< 0.001	1.467 (0.855, 2.079)	26.427	< 0.001

iER, intermittent energy restriction combined with HIIT group; cER, continuous energy restriction combined with HIIT group; ND, normal diet group combined with HIIT. BMI, body mass index; BF%, percentage body fat; WC, waist circumference; HC, hip circumference.

**TABLE 5:** Comparison of body composition and anthropometric parameters between groups.

	Group effect			IER *vs.* CER		IER *vs.* ND		CER v*s.* ND	
	F	*p*	η2*p*	MD	*p*	MD	*p*	MD	*p*
BW (kg)	0.623	0.544	0.043	–2.275	1.000	–3.495	0.746	–1.220	1.000
BMI (kg⋅m^–2^)	0.185	0.832	0.013	–0.276	1.000	–0.384	1.000	–0.108	1.000
BF%	3.082	0.062	0.180	–2.145	0.612	–3.605	0.110	–1.459	0.612
WC (cm)	0.805	0.457	0.054	–1.913	1.000	–3.173	0.315	–1.260	1.000
HC (cm)	1.778	0.188	0.113	–1.609	1.000	–3.323	0.151	–1.713	1.000

BMI, body mass index; BF%, percentage body fat; WC, waist circumference; HC, hip circumference; WHR, the waist-hip ratio.

The interaction of group and time for total lean mass [*F*_(2, 28)_ = 2.514, *p* = 0.099, η^2^*_*p*_* = 0.152], and variance test found no significant difference in the main effect of time or group factors (*p*>0.05). The interaction of group and time for WHR [*F*_(2, 28)_ = 2.212, *p* = 0.128, η^2^*_*p*_* = 0.136] was also not significant, and variance test found time factor has significant difference [*MD* = 0.008, 95% CI (0.003,0.013), *F*_(1, 14)_ = 11.224, *p* = 0.005], but no significant difference in the main effect of group factors (*p*>0.05).

### Blood lipids and cardiorespiratory fitness

After two-way ANOVA with repeated measures, the interaction of group and time for TG [*F*_(1_._240, 17_._364)_ = 2.922, *p* = 0.099, η^2^*_*p*_* = 0.173], HDL-c [*F*_(1_._432, 20_._053)_ = 1.857, *p* = 0.188, η^2^*_*p*_* = 0.117] and LDL-c [*F*_(1_._171, 16_._401)_ = 2.907, *p* = 0.103, η^2^*_*p*_* = 0.172]was not significant, besides TC [*F*_(2, 28)_ = 6.318, *p* = 0.005, η^2^*_*p*_* = 0.311] (Table 6). The main effect of time on TG [MD = -0.112 mmol⋅L^–1,^ 95% CI (-0.047, -0.177), *F*_(1, 14)_ = 13.611, *p* = 0.002, η^2^*p* = 0.493] and HDL-c [MD = 0.035 mmol⋅L^–1,^ 95%CI (0.068, 0.002), *F*_(1, 14)_ = 5.324, *p* = 0.037, η^2^*_*p*_* = 0.276] were significant, but there was no difference between groups (*p*>0.05). The main effect of time and group for LDL-c was not significant (*p*>0.05). After intervention TC was decreased significant [MD = -0.396 mmol⋅L^–1^, 95% CI (-0.233, -0.559), *F*_(1_._14)_ = 27.056, *p* < 0.001], and also between groups [*F*_(1_._311, 18_._349)_ = 4.716, *p* = 0.035, η^2^*_*p*_* = 0.252] (Table 6).

**TABLE 6:** Two-way ANOVA with repeated measures of blood lipids and CRF.

	Time effect			Group effect			Interaction effect		
	*F*	*p*	η^2^*p*	*F*	*p*	η^2^*p*	*F*	*p*	η^2^*p*
TC (mmol⋅L^–1^)	67.840	< 0.001	0.829	1.097	0.322	0.073	6.318	0.005	0.311
TG (mmol⋅L^–1^)	13.611	0.002	0.493	0.204	0.721	0.014	2.922	0.099	0.173
HDL-c (mmol⋅L^–1^)	5.324	0.037	0.276	0.541	0.588	0.037	1.857	0.188	0.117
LDL-c (mmol⋅L^–1^)	4.022	0.065	0.223	2.967	0.068	0.175	2.907	0.103	0.172
AV̇O_2m*ax*_ (L/min)	17.070	0.001	0.549	1.664	0.217	0.106	0.121	0.887	0.009
RV̇O_2m*ax*_ (mL/kg/min)	112.552	< 0.001	0.889	1.779	0.187	0.113	2.791	0.078	0.166

TC, total cholesterol; TG, triglyceride; LDL-c, low-density lipoprotein-cholesterol; HDL-c, high-density lipoprotein-cholesterol; AV̇O_2max_, absolute maximal oxygen uptake; RV̇O_2max_, relative maximal oxygen uptake.

After two-way ANOVA with repeated measures, the interaction of group and time for AV̇O_2m*ax*_ [*F*_(2, 28)_ = 0.121, *p* = 0.887, η^2^*_*p*_* = 0.009], RV̇O_2m*ax*_ [*F*_(2, 28)_ = 2.791, *p* = 0.078, η^2^*_*p*_* = 0.166] were not significant. After intervention RV̇O_2m*ax*_ [*MD* = 2.378 mL/kg/min, 95% CI (1.897, 2.858), *p* < 0.001, *F*_(1, 14)_ = 112.552, *p* < 0.001, η^2^*_*p*_* = 0.889] and AV̇O_2m*ax*_ [*MD* = 0.064 L/min, 95% CI (0.031, 0.097), *p* = 0.001, *F*_(1, 14)_ = 17.070, *p* < 0.001, η^2^*_*p*_* = 0.549] were significant, but there was no difference between groups (*p*>0.05) (Table 6).

## Discussion

We sought to examine if energy restriction diets combined with HIIT could significantly improve the effectiveness of weight loss compared to a normal diet with HIIT. We further compared the differences between an intermittent energy restriction diet and a continuous energy restriction diet when combined with HIIT. The main findings of this study were: (1) 4-week HIIT with or without dietary restriction intervention decreased body weight, BMI, BF%, WC, HC, and blood lipids, and improved CRF; (2) Body composition and anthropometric parameters showed greater reductions when the dietary restriction was combined with HIIT compared to the exercise intervention only group. There was also a greater reduction in the iER group compared to cER group, but there were no significant differences between groups; (3) Both iER, cER, and ND are similarly effective at reducing blood lipids and improving CRF.

The findings indicated that a short-term dietary restriction combined with an exercise intervention could be effective for weight loss. In terms of time effects, a 4-week dietary restriction combined with HIIT intervention could significantly reduce in body weight, BMI, BF%, WC, and HC. This phenomenon is consistent with the results of studies outlined previously in which dietary restriction was used only for 4-week (43). The present data showed that the reduction in body weight in the iER, cER, and ND groups was 4.6, 2.5, and 1.4, respectively. Other studies demonstrated 2.3 and 2.1 kg body weight reductions in sedentary population in IER only and CER only following 4-week (44, 45), which demonstrated that dietary restriction in combination with exercise could increase weight loss compared to a dietary restriction only or exercise only. These results were likely due to an increase in total energy consumption from a negative energy balance of reduced dietary calorie intake and increased physical activity expenditure, which, in combination, result in greater weight loss (22). In addition, a previous meta-analysis (46) demonstrated that it is necessary to include exercise in combination with diet to effectively elicit changes in body composition and biomarkers of metabolic issues, which is consistent with our results. Energy restriction could increase fat oxidation during exercise (47), increasing the utilization of fat in total energy expenditure. Under the conditions of energy restriction, fat usage during exercise could be increased, independent of changes in energy expenditure. Furthermore, there may be an underlying lipid metabolism, which may explain the differences between the coordination effects of the exercise (48). Previous investigations have demonstrated that detectible changes in exercise combined with dietary restriction can be used to reduce weight loss and sustain long-term weight loss compared to calorie restriction only (49, 50). In this study, we found that the improvement in body composition changes were small utilizing HIIT in isolation over the short term, but under the condition of calorie restriction, HIIT could lead to more fat loss.

Furthermore, we compared the changes between iER and cER, which represents the first randomized controlled trial, to the best of the investigative team’s knowledge to compare the effect of IER and CER on weight loss in overweight/obese adults when combined with HIIT exercise. The effects between IER and CER in weight reduction management are controversial. Previous studies have shown no significant differences in weight loss between IER and CER interventions (16, 51). Recent systematic reviews conclude that IER tended to decrease more body weight and fat mass in comparison to CER in overweight or obese subjects (52), it is superior in terms of improving lipid metabolism, resulting in a greater loss of body fat (8, 10). In the work of Xu et al. both IER and CER interventions could resulted in the body weight and BMI to be significantly lower in sedentary individuals, but without a significant difference between groups following a 4-week intervention (43). Our findings demonstrated that under the conditions of HIIT, the iER group had a greater reduction in body weight, BMI, BF%, WC, and HC compared to the cER group, but there was no significant difference between the groups. Therefore, we suggested that a longer trial, or an additional supplement, was needed to trigger the difference between iER and cER, and reduced more body weight. Additionally, the IER was easier to adhere to than CER when combined with exercise-trained (53).

Many studies, varying in intervention length, have shown that both IER and CER could reduce blood lipids, and no differences were observed between groups (52, 54). According to this study, two-way repeated measures ANOVA has shown a significant decrease in TC, TG, and HDL-c following the 4-week intervention, but there was no significant difference between iER, cER, and ND groups. Changes in lipidemia are responses to lipid metabolism, and previous studies have suggested that the underlying mechanisms responsible for the effects of energy restriction on lipidemia are related to metabolic adaptation (52). We suspected that there was no difference between IER and CER in combination with HIIT in blood lipids. Further research is needed to examine whether a difference would occur following a long-term intervention.

Weight loss is frequently accompanied by a reduction in lean body mass and muscle mass (17, 55), and a calorie-restricted diet has been observed to result in a decrease in absolute V̇O_2m*ax*_ (55, 56), a parameter of CRF. Our data found there is no change in lean mass and fat-free mass in response to iER, cER, and ND groups. These findings are in line with previous studies observing that IER and CER did not decrease fat-free mass in resistance-trained adults (53). These results are consistent with a previous study in mice, which demonstrated that HIIT rescued calorie restriction-mediated reductions in lean body mass and resting energy expenditure (57). We suggest that exercise attenuated the loss of lean mass irrespective of whether the IER or CER dietary intervention was followed. It can be inferred that the HIIT and energy restriction intervention is effective for promoting the loss of body fat without concurrent lean mass reduction. Previous studies have also shown that resistance training and aerobic training in conjunction with calorie restriction feeding could maintain lean mass and protect the absolute V̇O_2m*ax*_ (17, 58). As V̇O_2m*ax*_ is an important index to evaluate CRF levels, our findings indicate that energy restriction combined with HIIT improves cardiovascular health (59). Our findings also demonstrated a rise in absolute V̇O_2m*ax*_ and relative V̇O_2m*ax*_ in the iER, cER, and ND groups, but there were no differences between groups. The mechanisms underlying the effects of different dietary calorie restriction patterns on cardiorespiratory fitness need to be further investigated, possibly by genomic or metabolomic approaches, to investigate further the underlying biochemical principles involved in this type of activity. Future studies will need to consider the duration of the intervention, change the type of exercise, and expand the sample size of the study for further in-depth scientific exploration.

## Conclusion

(1) The combination of HIIT and energy restriction dietary are effective strategies for weight loss using a short-term intervention. Either iER or cER could reduce body weight, BMI, BF%, anthropometric parameters, and blood lipids while improving CRF in overweight/obese adults; (2) iER tends to be superior to cER in the reduction of body weight, BMI, BF%, WC, and HC; (3) There were no difference in the reduction in blood lipids and improvement in CRF between iER and cER.

## Data availability statement

The raw data supporting the conclusions of this article will be made available by the authors, without undue reservation.

## Ethics statement

The study design and experimental protocol were approved by the Nanjing Sports Institute Laboratory Ethics Committee (IRB No. RT202102). The patients/participants provided their written informed consent to participate in this study. Written informed consent was obtained from the individual(s) for the publication of any potentially identifiable images or data included in this article.

## Author contributions

RX and Y-TC designed the study. Y-XC collected and analyzed the data. RX and Y-XC undertook the data interpretation and manuscript preparation. RX and Y-TC contributed to the project administration. RX, Y-XC, and Y-QJ contributed to the supervision. All authors read and approved the final version of the manuscript.
